# Surgical nurses’ attitudes toward healthy nutrition and their e-health diet literacy: evidence from a descriptive and correlational cross-sectional study in Afyonkarahisar, Turkey, 2025

**DOI:** 10.1186/s12912-025-04113-6

**Published:** 2025-11-27

**Authors:** Büşra Koca, Cahide Çevik

**Affiliations:** 1 Isparta City Hospital, Isparta, Turkey; 2 Faculty of Health Sciences, Public Health Nursing Department, Afyonkarahisar Health Sciences University, Afyonkarahisar, Turkey

**Keywords:** Attitude, Diet, Healthy, Literacy, Nurses, Nutrition

## Abstract

**Background:**

Nurses working in surgical units—where acute clinical situations are frequently encountered—are particularly at greater risk as a result of irregular schedules and increased workload. These professional conditions can negatively impact dietary habits, which are among the fundamental components of a healthy lifestyle. In mitigating the adverse effects of working conditions on nutritional behavior, nurses’ attitudes toward healthy nutrition emerge as a significant determinant.

**Objective:**

The aim of this study is to examine the attitudes of surgical nurses toward healthy nutrition, their levels of e-health diet literacy, and the relationship between these two variables.

**Method:**

This descriptive and correlational cross-sectional study was conducted between February 15 and May 15, 2024, among 376 nurses working in the surgical clinics of one secondary- and one tertiary-level hospital using a convenience sampling method. Data were collected using a Personal Information Form, the Healthy Nutrition Attitude Scale, and the E-Health Diet Literacy Scale. Data analysis was performed using IBM SPSS Version 25.0.

**Results:**

The mean age of the surgical nurses was 32.32 ± 6.20 years. The mean score for e-health diet literacy among the nurses was 39.00 ± 8.33, while the mean score for healthy nutrition attitude was 71.34 ± 12.01. It was found that 61.9% of the nurses enjoyed consuming fast food products, and 69.7% reported searching for healthy nutrition information on unofficial sources such as blogs and advertisements, although the frequency varied. A positive correlation was identified between e-health diet literacy and healthy nutrition attitude among the nurses (*r* = 0.469, *p* < 0.001). In addition, e-health diet literacy was found to be a positive predictor of healthy nutrition attitude (B = 0.676, *p* = 0.000).

**Conclusion:**

In conclusion surgical nurses demonstrate a positive attitude toward healthy nutrition; however, their level of e-health diet literacy is less than moderate. Furthermore, it can be concluded that as levels of e-health diet literacy increase, attitudes toward healthy nutrition become more positive. It is therefore recommended that institutional strategies be developed to improve nurses’ access to healthy food during shifts and that training programs be implemented to strengthen their e-health nutrition literacy.

## Background

Nursing encompasses various fields of practice that involve providing care for individuals across all age groups [1], whether healthy or ill [2], and includes roles in advocacy, health education [3], research, and policy development [4, 5]. Among these fields, surgical nursing represents a specialized area that requires advanced knowledge and clinical competence to provide holistic care for patients undergoing surgical procedures throughout the preoperative, intraoperative, and postoperative phases [6]. Surgical nurses typically work in operating rooms, surgical wards, postoperative care units, surgical intensive care units, and ambulatory surgery centers. Ferramosca et al. (2023) reported that nurses working in medical-surgical units experience high levels of physical, mental, and emotional workload, influenced by patient complexity, specialty requirements, unplanned activities, and extensive documentation [7]. These factors are particularly pronounced in surgical settings, where the demanding nature of perioperative care and the need for continuous attention to patient safety further intensify the workload and physical strain on nurses. These work environments demand intense focus, quick reflexes, and a high level of physical endurance [8, 9]. Therefore, ensuring appropriate working conditions that protect the physical health and support the psychological well-being of surgical nurses is essential to sustaining their performance and improving their quality of life. Factors such as long working hours, night shifts, inadequate staffing, and heavy workloads negatively affect their quality of life [10]. Kızıl Toğaç and Yılmaz (2024) reported a significant negative association between workload and quality of life among nurses working in surgical units. The researchers observed that increasing workload adversely affected nurses’ physical and mental health. The authors further emphasized the necessity of developing effective strategic plans aimed at reducing nurses’ workload and enhancing their overall health status [11]. The demanding working conditions of surgical nurses are an important factor that directly affects not only their quality of life but also their eating habits. Long working hours, stress, physical fatigue, insufficient sleep, and irregular meals make it difficult for nurses to develop healthy eating habits. This situation may negatively impact their overall health, job performance, and relationships with patients [10, 12]. Therefore, to reduce nutrition-related risks, it is crucial for nurses to develop a positive attitude toward healthy eating. Nutritional attitude, in turn, is closely associated with the level of nutritional knowledge [13, 14]. Nowadays, the way people access information has changed, and the internet has become a primary source of nutrition-related knowledge. However, the ability to access accurate information and to use it in making healthy decisions is closely linked to individuals’ level of nutrition literacy [15]. Nutrition literacy refers to the capacity to obtain, process, and understand the knowledge and skills necessary for making appropriate decisions about nutrition [16]. In this context, e-health nutrition literacy refers to the ability to read, understand, evaluate, and apply information obtained from electronic resources in order to adopt healthy eating behaviors [17]. Zigdon, Zigdon, and Moran (2020) reported that, although nurses are graduates of health sciences, they frequently search the internet for medical and health-related information to meet their personal needs [18]. This finding highlights the increasing influence of digital platforms in shaping health behaviors and dietary habits. However, the literature on the relationship between surgical nurses’ e-health diet literacy and their attitudes toward healthy nutrition is limited. Furthermore, the relationships of healthy nutrition attitudes with e-health diet literacy, as well as with demographic and work-related variables, have not been clearly established. Therefore, the aim of this study is to investigate the relationship between surgical nurses’ e-health diet literacy levels and their healthy nutrition attitudes, and to evaluate the associations of e-health diet literacy together with demographic and work-related variables with healthy eating attitudes. Demonstrating this relationship may offer valuable insights into how surgical nurses integrate healthy nutrition information acquired from digital sources into their daily lives. In this way, the study may contribute to the existing body of knowledge regarding the link between access to information and the development of healthy lifestyle behaviors among nurses. Furthermore, the findings of this study are expected to guide the strategic development of nutrition education and digital health diet literacy training programs targeted at nurses.

This study seeks to answer the following research questions:

### RQ1

What are the mean scores of surgical nurses on the Healthy Nutrition Attitude Scale?

### RQ2

What are the mean scores of surgical nurses on the E-Health Diet Literacy Scale?

### RQ3

What are the frequencies and percentages of surgical nurses’ responses to the items on the Healthy Nutrition Attitude Scale and the E-Health Diet Literacy Scale?

### RQ4

 Is there a statistically significant relationship between e-health diet literacy and healthy nutrition attitudes among surgical nurses?

### RQ5

To what extent do e-health diet literacy, age, gender, and work schedul predict surgical nurses’ healthy eating attitudes?

## Method

### Design

This study employed a descriptive and correlational cross-sectional design. Specifically, the descriptive component aimed to determine surgical nurses’ attitudes toward healthy nutrition and their levels of e-health diet literacy, whereas the correlational component examined the relationship between these two variables. The study was conducted between February 15 and May 15, 2024, among nurses employed in the surgical clinics of two hospitals: a secondary-level public hospital and a tertiary-level university hospital.

### Study sample

The population of the study consisted of 520 nurses working in the surgical clinics of the two hospitals. While the study aimed to include all nurses working in the surgical units of the two hospitals, a convenience sampling approach was adopted, including nurses who met the inclusion criteria and voluntarily agreed to participate. A total of 144 nurses were not included in the study as they did not meet the inclusion criteria, were on leave during the data collection period, or declined to participate. Accordingly, a total of 376 nurses were included, covering 72% of the target population. Some of these nurses work permanent day shifts, some work only night shifts (4:30 p.m.–8:30 a.m.), and others work in a rotating shift system that includes both day and night shifts.

### Inclusion criteria

The inclusion criteria were nurses with more than one year of professional experience in surgical units (including surgical intensive care, wards, and operating rooms), who did not report any psychiatric disorders that could affect the study, and who voluntarily agreed to participate. The inclusion criterion requiring participants to have worked in surgical clinics for at least one year was established to ensure their adaptation to the intensive and demanding work pace of these settings, thereby enabling a more accurate evaluation of the relationship between healthy eating literacy and healthy eating attitudes under such conditions. The inclusion criterion related to the absence of psychiatric disorders was determined to ensure that participants could provide reliable self-reports and consistent responses regarding attitudes and perceptions.

### Exclusion criteria

Nurses who had less than one year of professional experience, were on leave during the data collection period, declined to participate, or reported having a psychiatric disorder that could affect the study were excluded.

### Data collection tools and procedure

Data were collected using the “Personal Information Form,” the “Healthy Nutrition Attitude Scale,” and the “e-Health Diet Literacy Scale.” Data collection was conducted face-to-face by the researcher through workplace visits with the nurses. The data collection process took approximately 15 min. The scales were not pilot-tested prior to data collection, as they had been previously validated.

### Personal information form

This form, developed by the researchers, included questions regarding personal characteristics such as age, gender, educational status, unit of employment, and work schedule.

### Healthy nutrition attitude scale

The scale was developed by Tekkurşun Demir and Cicioğlu (2019), and its validity and reliability analyses were conducted [19]. The scale explains 57.79% of the total variance. The factor loadings of the factors in the measurement tool were found to be higher than 0.40, with the factor loadings of each item ranging between 0.41 and 0.95. Subsequently, the four-factor structure of the scale was confirmed by confirmatory factor analysis. The test–retest reliability coefficients were calculated as 0.81, 0.79, 0.68, and 0.80, respectively. It consists of 21 items and four subdimensions: knowledge about nutrition, feelings toward nutrition, positive nutrition, and poor nutrition. The nutrition knowledge sub-dimension consists of items 1, 2, 3, 4, and 5; the nutrition-related emotion sub-dimension consists of items 6, 7, 8, 9, 10, and 11; the positive nutrition sub-dimension consists of items 12, 13, 14, 15, and 16; and the poor nutrition sub-dimension consists of items 17, 18, 19, 20, and 21. The nutrition knowledge dimension includes items that assess individual awareness and understanding of healthy nutrition. The nutrition-related emotion dimension includes items that capture positive or negative feelings toward nutrition. The positive nutrition dimension includes items that indicate choices and habits that behaviorally support healthy eating. The poor nutrition dimension consists of items that represent behavioral tendencies or habits related to unhealthy eating. The items are rated on a 5-point Likert-type scale. The response options for the positive items in the scale are ‘Strongly Disagree,’ ‘Disagree,’ ‘Neutral,’ ‘Agree,’ and ‘Strongly Agree.’ Positive attitude items are scored from 1 to 5, while negative attitude items are reverse-scored from 5 to 1. The score range of the scale is 21–105. Scores are interpreted as follows: 21 points = very low, 23–42 = low, 43–63 = moderate, 64–84 = high, and 85–105 = ideally high level of healthy nutrition attitude. In this study, the Cronbach’s alpha coefficient of the scale was calculated as 0.89.

### e-Health diet literacy scale

The scale was originally developed by Van Duong et al. (2020) [20], and its Turkish validity and reliability study was conducted by Onbaşı and Türker [21]. The factor loadings of the items in each dimension were above 0.40, and the total variance explained by the scale was determined as 73.5%. The Cronbach’s alpha internal consistency coefficient of the scale was found to be 0.77. The correlation coefficient of the test–retest scores of the scale was found to be 0.98. Four of the items were adapted from the Digital Healthy Diet Literacy Scale. The e-Health Diet Literacy Scale consists of five factors: finding e-health diet information, understanding e-health diet information, evaluating e-health diet information, and applying e-health diet information, with a total of 15 items. Higher scores indicate higher levels of e-health diet literacy. Confirmatory factor analysis supported the five-factor model of the scale. In the scoring of the scale: responses to the first sub-dimension are scored as never = 1, a few times a year = 2, a few times a month = 3, a few times a week = 4, and every day = 5; responses to the second sub-dimension are scored as correct = 5 and incorrect or don’t know = 1; responses to the third sub-dimension range from strongly disagree = 1 to strongly agree = 5; responses to the fourth sub-dimension range from never = 1 to always = 5; and responses to the fifth sub-dimension are scored from very difficult = 1 to very easy = 4. The total score of all responses constitutes the overall scale score. The maximum possible score is 71. In the present study, the Cronbach’s alpha coefficient of the scale was calculated as 0.69.

### Ethics

The study was carried out in accordance with the ethical principles of the Declaration of Helsinki. Ethical approval for the study was obtained from the Afyon Kocatepe University Health Sciences Scientific Research and Publication Ethics Committee (Date: 14.02.2024, No: 2024/05). Informed consent was obtained from all participating nurses, and the confidentiality and anonymity of participants and their data were strictly maintained throughout the research process.

### Statistical analysis

Data analysis was performed using IBM SPSS Statistics for Windows, Version 25.0. To assess the normality of the data distribution, skewness–kurtosis values and the Kolmogorov–Smirnov test were used. Since the skewness and kurtosis coefficients were within the range of − 2 to + 2, the assumption of normality was accepted. Descriptive statistics for the scales were presented using arithmetic means and standard deviations. The relationship between e-health diet literacy and healthy nutrition attitude was tested using Pearson correlation analysis. Simple linear regression analysis was performed to examine the predictive role of e-health diet literacy in healthy nutrition attitude. In addition, to identify other predictors of healthy nutrition attitude, a multiple regression model was constructed with e-health diet literacy, age, gender, and work schedule as independent variables, and healthy nutrition attitude as the dependent variable. The independent variables were entered into the analysis simultaneously using the enter method. Dummy variables were created for the categorical independent variables, with ‘female’ as the reference category for gender and ‘permanent daytime work’ as the reference category for work schedule. A p-value of < 0.05 was considered statistically significant in all analyses.

## Results

The mean age of the participating nurses was 32.32 ± 6.20 years, and their average length of professional experience was 9.62 ± 6.83 years. Among the nurses, 58.5% (*n* = 220) were female, and 41.5% (*n* = 156) were male. Regarding educational background, 79% (*n* = 297) held a bachelor’s degree, 13.3% (*n* = 50) had an associate degree, 4.3% (*n* = 16) had a postgraduate degree, and 3.5% (*n* = 13) had completed high school (Table 1).

In terms of the type of institution, 62.8% (*n* = 236) were working in state hospital, while 37.2% (*n* = 140) were employed in university hospital. Distribution by unit showed that 42.6% (*n* = 160) worked in intensive care units, 37% (*n* = 139) in wards, and 20.5% (*n* = 77) in operating rooms. Regarding work schedules, 65.2% (*n* = 245) reported working in shifts, 17.8% (*n* = 67) worked only day shifts, and 17% (*n* = 64) worked only night shifts (Table 1).

In response to the question “Do you think you are able to eat regularly during your working hours?”, 81.6% (*n* = 307) answered “no” and 18.4% (*n* = 69) answered “yes.”

The mean e-health diet literacy score of the surgical nurses was 39.00 ± 8.33, while the mean healthy nutrition attitude score was 71.34 ± 12.01. These findings highlight a clear discrepancy between nurses’ high attitudes toward healthy nutrition and their below-moderate level of digital diet literacy, indicating that positive nutritional attitudes are not fully reflected in their digital health competencies.

**Table 1:** Sociodemographic characteristics of the nurses participating in the study (*N* = 376)

	*n*	%
**Gender**		
Female	220	58.5
Male	156	41.5
**Education level**		
High school	13	3.5
Associate degree	50	13.3
Bachelor’s degree	297	79
Postgraduate degree	16	4.3
**Type of hospital**		
University hospital	140	37.2
State hospital	236	62.8
**Unit of employment**		
Surgical Ward	139	37
Surgical Intensive care	160	42.6
Operating room	77	20.5
**Work Schedule**		
Permanent day shift	67	17.8
Permanent night shift	64	17
Shift work	245	65.2
**Do you think you are able to maintain a regular eating routine during your working hours?**		
Yes	69	18.4
No	307	81.6
	**Mean** ± **SD**	
Age	32.32 ± 6.20	
Professional experience	9.62 ± 6.83	

Analysis of the nurses’ responses to the Healthy Eating Attitude Scale revealed that a considerable proportion of participantsenjoyed consuming sugary foods (57.7%), fast-food products (61.9%), delicatessen items (47.4%), fried foods (54.5%), and syrupy desserts (67.6%). Additionally, some nurses did not regularly consume main meals (21%), had insufficient intake of fruits (37,5%) and vegetables (29,3%), and occasionally skipped meals (20.2%) (Table 2).

The findings regarding the e-health nutrition literacy of surgical nurses are presented in Table 3. A considerable proportion of the nurses (79%) reported that they searched for information on healthy nutrition via the internet. Notably, most of them used unofficial sources, including advertisements and blogs (69.8%). Regarding knowledge items, the statements related to starchy foods (67.3%), sweet fruits (69.7%), and egg yolks (63.3%) were largely evaluated as incorrect or uncertain. In contrast, the majority (75.3%) correctly identified the statement “food additives are harmful to the human body.” Most participants considered online information provided by official institutions (71.5%) and healthcare professionals (73.2%) to be more reliable than that from other sources. However, a considerable proportion of participants reported that they neither shared incorrect information encountered online (43.9%) nor discussed such information with professional healthcare workers (37.8%). Moreover, many nurses indicated that finding (50%), understanding (46.3%), evaluating the validity of (56.1%), and applying online information on healthy nutrition to daily life (65.2%) was difficult.

The correlation analysis revealed a moderate positive relationship between surgical nurses’ healthy nutrition attitudes and their e-health diet literacy scores (*r* = 0.469, *p* < 0.001) (Table 4). A simple regression analysis showed that e-health diet literacy significantly predicted healthy nutrition attitude (F = 105.24, *p* < 0.001), explaining 21.8% of its variance. Specifically, a one-unit increase in e-health diet literacy was associated with a 0.68-unit increase in healthy nutrition attitude.

When age, gender, and work schedule were added to the model, the explained variance increased to 30.5% (F = 33.90, *p* < 0.001) (Table 5). In this extended model, higher e-health diet literacy (B = 0.52, *p* < 0.001), older age (B = 0.23, *p* < 0.001), and female gender (B = 4.56, *p* < 0.001) were positive predictors of healthy nutrition attitude, whereas night shifts (B = − 6.25, *p* = 0.001) and shift work (B = − 4.11, *p* = 0.004) were negative predictors.

**Table 2:** Response distribution of surgical nurses to the healthy nutrition attitude scale

	Strongly disagree		Disagree		Neutral		Agree		Strongly agree	
	N	%	n	%	n	%	n	%	n	%
**Items**										
1. I know the benefits of healthy eating.	3	0.8	14	3.7	39	10.4	187	49.7	133	35.4
2. I know which foods contain protein.	2	0.5	10	2.7	71	18.9	202	53.7	91	24.2
3. I know which foods contain carbohydrates.	2	0.5	10	2.7	68	18.1	204	54.3	92	24.5
4. I know which foods contain vitamins/minerals.	3	0.8	12	3.2	91	24.2	191	50.8	79	21.0
5. I know what healthy foods are.	1	0.3	12	3.2	58	15.4	192	51.1	113	30.1
6. I feel happy when I consume sugary foods (chocolate, cake, biscuits, etc.).	6	1.6	57	15.2	96	25.5	**146**	**38.8**	**71**	**18.9**
7. I enjoy eating fast food products (hamburgers, pizza, etc.).	12	3.2	61	16.2	70	18.6	**169**	**44.9**	**64**	**17.0**
8. I enjoy eating delicatessen products (salami, sausage, sucuk, etc.).	31	8.2	85	22.6	82	21.8	**121**	**32.2**	**57**	**15.2**
9. I like eating fried foods.	23	6.1	56	14.9	92	24.5	**129**	**34.3**	**76**	**20.2**
10. I do not enjoy eating fruit.	112	29.8	143	38.0	46	12.2	45	12.0	30	8.0
11. I feel happy when I consume syrupy desserts (baklava, künefe, etc.).	11	2.9	40	10.6	71	18.9	**154**	**41.0**	**100**	**26.6**
12. I regularly eat main meals (breakfast, lunch, and dinner).	**14**	**3.7**	**65**	**17.3**	131	34.8	120	31.9	46	12.2
13. I drink at least 1.5 L of water per day.	10	2.7	46	12.2	103	27.4	119	31.6	98	26.1
14. I consume vegetables at least three times a week.	**15**	**4.0**	**95**	**25.3**	110	29.3	102	27.1	54	14.4
15. I consume fruit regularly.	**27**	**7.2**	**114**	**30.3**	99	26.3	91	24.2	45	12.0
16. I eat foods containing protein (meat, milk, eggs, etc.) every day.	8	2.1	69	18.4	102	27.1	141	37.5	56	14.9
17. I skip main meals.	63	16.8	119	31.6	118	31.4	**67**	**17.8**	**9**	**2.4**
18. I eat junk food (chips, chocolate, biscuits, etc.) every day.	86	22.9	153	40.7	77	20.5	49	13.0	11	2.9
19. I drink at least one glass of carbonated/soft drinks every day.	132	35.1	141	37.5	45	12.0	45	12.0	13	3.5
20. I eat on the go.	86	22.9	126	33.5	98	26.1	58	15.4	8	2.1
21. I usually replace my main meals with foods like cake or biscuits.	120	31.9	147	39.1	71	18.9	33	8.8	5	1.3

Bold values highlight response categories that are considered noteworthy for each item, either because they represent the highest percentages or because they reflect response tendencies of particular interpretive importance

**Table 3:** Distribution of surgical nurses’ responses to the e-Health diet literacy scale items

	Never		A few times a year		A few times a month		A few times a week		Every day	
	N	%	n	%	n	%	n	%	n	%
**In the past year, how often have you…**										
…searched for information about healthy eating on the internet?	79	21.0	**115**	**30.6**	**116**	**30.9**	**52**	**13.8**	**14**	**3.7**
…searched for information about healthy eating on institutional/official websites such as public research institutes, government agencies, the Ministry of Health, or hospital websites?	128	34.0	**144**	**38.3**	**73**	**19.4**	**21**	**5.6**	**10**	**10**
…searched for information about healthy eating on unofficial websites such as advertisements and blog pages?	114	30.3	**107**	**28.5**	**100**	**26.6**	**36**	**9.6**	**19**	**5.1**
**Below are some statements you may frequently encounter on the internet. Do you think these statements are true?**	**True**		**False / I don’t know**							
Avoiding starchy foods may lead to weight loss.	123	32.7	**253**	**67.3**						
Sweet fruits should be avoided to maintain blood sugar within the correct range.	114	30.3	**262**	**69.7**						
Individuals with high cholesterol should avoid eating egg yolks.	138	36.7	**238**	**63.3**						
Food additives are harmful to the human body.	**283**	**75.3**	**93**	**24.7**						
**Please indicate your level of agreement with the following statements**:	**Strongly disagree**		**Disagree**		**Neutral**		**Agree**		**Strongly agree**	
Online healthy eating information provided by official institutions is more reliable than that provided by unofficial sources.	16	4.3	34	9.0	57	15.2	**175**	**46.5**	**94**	**25.0**
Online healthy eating information created by dietitians and/or other healthcare professionals is more reliable than information created by non-professionals.	12	3.2	34	9.0	55	14.6	**159**	**42.3**	**116**	**30.9**
**How often do you….**	**Never**		**Rarely**		**Sometimes**		**Often**		**Always**	
…share your personal opinion online about incorrect information related to healthy eating that you come across on the internet?	**165**	**43.9**	91	24.2	95	25.3	19	5.1	6	1.6
… share nutrition-related information found on the internet with a professional healthcare worker?	**142**	**37.8**	122	32.4	82	21.8	24	6.4	6	1.6
**Please indicate your opinion for the following statements in a scale ranging from very difficult to very easy.**	**Very Difficult**		**Somewhat Difficult**		**Somewhat Easy**		**Very Easy**			
Finding reliable and accurate information about healthy eating on the internet…	**61**	**16,2**	**127**	**33,8**	133	35.4	55	14.6		
Understanding the information and dietary recommendations related to healthy eating found on the internet…	**51**	**13,6**	**123**	**32,7**	145	38.6	57	15.2		
Deciding whether the healthy eating information found on the internet is applicable to you…	**80**	**21,3**	**131**	**34,8**	131	34.8	34	9.0		
Applying the healthy eating information you obtain from the internet to your daily life in order to eat better…	**98**	**26,1**	**147**	**39,1**	105	27.9	26	6.9		

Bold values highlight response categories that are considered noteworthy for each item, either because they represent the highest percentages or because they reflect response tendencies of particular interpretive importance

**Table 4:** Correlation analysis results between attitudes toward healthy nutrition and e-Health diet literacy

Variables	Mean	SS	1	2
1. Healthy Nutrition Attitude	71.34	12.01	1	
2. e-Health Diet Literacy	39.01	8.33	0.469*	1

*:p < 0.001

**Table 5:** Predictors of surgical nurses’ healthy nutrition attitudes

		Lower Bound	Upper Bound							
**Model 1**	**B**	**95.0% Confidence Interval**		**Beta**	**t**	***p***	**Zero-order**	**Partial**	**Part**	**VIF**
Constant	44.966	39.798	50.134		17.109	0.000				
e-Health Diet Literacy	0.676	0.547	0.806	0.469	10.259	0.000	0.469	0.469	0.469	1.000
*F = 105.244; Adj.R*^*2*^ *= 0.218; SEE = 10.63; Durbin-Watson = 2.049 B: Non-standardized coefficient. Beta: standardized coefficient*										
**Model 2**	**B**	**95.0% Confidence Interval**		**Beta**	**t**	**p**	**Zero-order**	**Partial**	**Part**	**VIF**
Constant	44.673	36.543	52.802		10.806	0.000				
e-Health Diet Literacy	0.524	0.389	0.660	0.363	7.594	0.000	0.469	0.367	0.327	1.235
Age	0.225	0.058	0.393	0.116	2.645	0.009	0.171	0.136	0.114	1.045
**Gender**										
Female	4.558	2.157	6.960	0.187	3.732	0.000	0.408	0.190	0.161	1.357
**Work schedule**										
Permanent night shift	-6.253	-9.988	-2.519	-0.196	-3.293	0.001	-0.236	-0.169	-0.142	1.909
Shift work	-4.109	-6.905	-1.313	-0.163	-2.890	0.004	-0.007	-0.149	-0.124	1.720

*F = 33.886; Adj.R*^*2*^ *= 0.305; SEE = 10.02; Durbin-Watson = 2.017 B: Non-standardized coefficient. Beta: standardized coefficient*

## Discussion

In this study, the relationship between surgical nurses’ e-health diet literacy and their healthy nutrition attitudes was examined. In addition, the associations of certain demographic and work characteristics with healthy nutrition attitudes were evaluated alongside e-health diet literacy. In this study, a statistically significant and positive relationship was found between e-health diet literacy and healthy nutrition attitude. However, the descriptive findings showed that the healthy nutrition attitude levels of surgical nurses were high, while their e-health diet literacy levels were slightly below the moderate level. At first glance, this may seem contradictory, but in fact, different levels of mean scores do not eliminate the positive relationship between the two variables. That is, as e-health diet literacy increases in the sample, healthy nutrition attitudes also increase; however, the overall means indicate different levels between the variables. In addition, it was found that age, gender, and working pattern, as well as e-health diet literacy, are important variables affecting healthy eating attitude. Therefore, although e-health diet literacy is an important determinant, it is not sufficient to explain the attitude level on its own; this also explains the differences in the mean levels.

Nurses’ positive attitudes toward healthy nutrition are consistent with the high level of health awareness expected from their professional roles. Analysis of nurses’ responses regarding healthy nutrition revealed that they are aware of the basic components of nutrition (proteins, carbohydrates, vitamins, and minerals) and report positive attitudes toward healthy habits such as regular consumption of main meals, adequate daily water intake, and frequent consumption of vegetables and fruits. Albert et al. (2014) [22] reported that clinical nurses exhibited high levels of healthy nutrition attitudes. Similarly, Sezen (2024) [23] found that nurses demonstrated positive attitudes toward healthy nutrition.

However, it is noteworthy that interest in unhealthy food choices, such as fast food, processed meat products, and fried foods, continues in our study. This finding can be interpreted as a reflection of the inconsistency between knowledge, attitude, and behavior. Indeed, previous research by Ling et al., Navruz Varlı and Mortaş, Clark et al., and Gupta et al. demonstrated that although health professionals often possess sufficient knowledge, environmental and occupational factors—such as shift work, time constraints, and limited food access—may restrict their ability to maintain healthy eating behaviors. Therefore, our findings highlight that in order to translate positive attitudes into behavior, not only individual awareness but also institutional and environmental regulations are necessary [24–27].

Furthermore, another noteworthy finding is that e-health diet literacy remained below the moderate range. Although nurses demonstrated strong awareness of health-related behaviors, their ability to access, use, and evaluate digital nutrition information appeared limited. This may be attributed to restricted access to digital resources, lack of confidence, or insufficient training. Kaçar and Türker (2024) observed that healthcare workers had low e-health diet literacy [28]. Collectively, these findings suggest that e-health diet literacy should be improved not only among surgical nurses but also across healthcare professionals in general. Therefore, implementing training programs to strengthen digital health literacy may contribute to enhancing nurses’ healthy nutrition attitudes.

Given this, the distribution of nurses’ responses to the e-health diet literacy scale revealed that most nurses search for healthy nutrition information on the internet. These findings reveal that information-seeking behavior in digital environments is common among nurses. This finding is consistent with previous research. Keshavarz et al. (2024) also reported that the frequency of obtaining information via the internet was high among health professionals [29]. It was also observed that the number of nurses who reported seeking information from informal sources (e.g., advertisements, blogs) was quite high. This suggests that their ability to critically evaluate the accuracy of information on these platforms is limited. Indeed, this finding is consistent with the results of Keshavarz et al. (2024), which also demonstrated that health professionals’ ability to critically evaluate the accuracy of digital information is limited [29].

Considering the abundance of misleading or controversial information about food on the internet, the majority of nurses correctly responded to the statement “food additives are harmful to the human body.” Nevertheless, the relatively high percentages of “incorrect/don’t know” responses regarding starchy foods and sweet fruits (67.3% and 69.7%) point to notable knowledge gaps in these specific nutrition topics. Given that nurses play a central role in providing patient education on nutrition, ensuring that they possess accurate and up-to-date information is essential. Future research should identify nurses’ knowledge gaps in nutrition and guide the development of educational programs accordingly.

Most nurses reported trusting online nutrition information provided by official institutions and recognized experts. This finding indicates that healthcare professionals tend to obtain nutrition information from reliable sources in digital environments.

However, it is noteworthy that approximately one-third of nurses stated that they do not share incorrect information obtained from the internet with their professional colleagues, and about half expressed hesitation in sharing their personal opinions about misinformation encountered online. Such behavior may result from factors such as professional trust dynamics, limited time, insufficient digital literacy, or uncertainty regarding the accuracy of available information [30].

When evaluating the ease of accessing reliable and accurate nutrition information online, approximately half of the nurses found it “difficult,” while the other half found it “easy.” This finding indicates that the presence of unreliable information on the internet poses a challenge for healthcare professionals. Consistent with this finding, Keshavarz et al. (2024) reported that healthcare professionals experience difficulties in assessing the quality and accuracy of online information and in managing inappropriate digital content [29].

Furthermore, many nurses reported difficulties in adapting nutrition information obtained from the internet to their daily lives. This raises questions about the practical applicability of digital health information. Future research should explore in greater depth how nurses integrate nutrition information acquired from digital sources into their personal routines and the specific challenges they face in doing so.

The present study demonstrated that surgical nurses with higher e-health diet literacy displayed more positive healthy nutrition attitudes. Both correlation and regression analyses confirmed a significant positive relationship between these two variables, indicating that e-health diet literacy is a strong predictor of healthy nutrition attitudes. This finding suggests that improving nurses’ digital competencies may contribute to healthier dietary behaviors. Similar to our results, Kaçar and Türker (2024) reported that e-health literacy significantly influenced individuals’ eating habits and anthropometric parameters among healthcare professionals [28]. Their study supports the idea that digital literacy contributes to better dietary awareness and healthier food choices. Likewise, Yeşildemir (2023) found a positive association between e-health literacy and sustainable healthy eating behaviors in adults, emphasizing its critical role in developing long-term healthy nutrition practices [31]. Together, these studies reinforce the evidence that higher e-health literacy facilitates positive nutrition-related attitudes and behaviors.

However, to generalize these findings, further studies with larger sample sizes are required. Moreover, it is important to emphasize that digital literacy should not be limited to the process of obtaining information but should also include the critical evaluation of its accuracy and the ability to access reliable data. Therefore, increasing digital literacy training for nurses to effectively utilize digital health information is a crucial step in promoting healthy eating habits.

Alongside e-health diet literacy, demographic factors were also found to influence nurses’ healthy nutrition attitudes. The analysis revealed that age was a positive predictor of healthy nutrition attitudes. As individuals grow older, they may attach greater importance to maintaining their health and improving their quality of life. Increased health awareness, accumulated life experience, and a heightened perception of chronic disease risks may contribute to this tendency. Karagöz et al. (2023) also reported a positive association between age, healthy nutrition attitudes, and health-related quality of life [32].

Women exhibited more positive attitudes toward healthy nutrition than men. The differences in personality traits between women and men may have contributed to this result. Women tend to be more sensitive and detail-oriented regarding their health, which may make them more attentive to seeking nutrition-related information [33], applying it, and developing healthy dietary attitudes. Schulman and Karney (2023) as well as Akduran et al. (2003) reported that women demonstrated more positive nutrition attitudes than men, supporting the gender-related differences observed in this study [34, 35]. Although the samples in these studies did not consist of surgical nurses, their findings are consistent with the present results.

In addition to demographic factors such as age and gender, occupational characteristics were also found to influence nurses’ healthy nutrition attitudes. Night shift and shift work were negative predictors of healthy nutrition attitude, indicating that nurses working regular day shifts demonstrated more positive attitudes toward healthy nutrition. Greater access to healthy food sources such as canteens, restaurants, or home-cooked meals available during daytime hours, together with more regular daily routines, relatively lower stress levels [36, 37], and stronger social support networks [37], may contribute to healthier eating behaviors. Nurses working at night, on the other hand, are often forced to rely on ready-made, packaged, and high-calorie foods due to limited options. Therefore, institutional regulations and training programs are needed to support healthy eating behaviors among nurses working shifts. Boivin et al. (2022) reported that shift work disrupts the circadian rhythm, and that sleep deprivation and melatonin suppression negatively affect physiological functions [38]. These disruptions are likely to influence eating habits negatively. Şimşekoğlu and Mayda (2016) also found that shift work adversely affected nurses’ healthy lifestyle behaviors and that healthy eating received less priority [39]. These findings are consistent with the negative relationship between shift work and nutrition.

## Limitations of the study

This study was limited to surgical nurses working in two hospitals; therefore, the results cannot be generalized to nurses employed in different regions or institutions. Another limitation of the study is the use of a non-probabilistic sampling method. In addition, because there are no other studies examining the same two variables, the scope of the discussion was restricted. Furthermore, nutritional disorders and special dietary practices were not assessed in this study. Given that these factors may influence nutritional attitudes, this was acknowledged as a limitation of the study.

## Conclusion

Based on the findings of this study, it can be concluded that nurses with higher skills in evaluating and utilizing nutrition information presented in digital environments tend to have more positive attitudes toward healthy nutrition. In addition to e-health diet literacy, factors such as age, female gender, and working regular day shifts were also found to positively predict healthy nutrition attitudes. In shift work, especially for nurses working night shifts, access to healthy food options may be limited. Therefore, it is important for institutional policies to be adjusted to support nurses’ eating habits. Healthy food options, particularly for those working night shifts, should be made more accessible. Administrative measures should be considered to reduce the workload of surgical nurses and to promote healthier lifestyle behaviors. Furthermore, implementing training programs aimed at enhancing digital healthy eating literacy among surgical nurses should be encouraged. These programs would help individuals improve their ability to obtain, evaluate, and apply accurate nutrition information in the digital environment. Awareness campaigns that encourage the use of reliable online sources for nutrition should be expanded, and nurses’ awareness of misleading and unreliable sources should be increased.

## Data Availability

The data that support the findings of this study are available on request from the corresponding author.

## Abbreviations

IBM: International Business Machines
SPSS: Statistical Package for the Social Sciences
